# Pediatric Obesity-Related Asthma: The Role of Nutrition and Nutrients in Prevention and Treatment

**DOI:** 10.3390/nu13113708

**Published:** 2021-10-21

**Authors:** Valeria Calcaterra, Elvira Verduci, Michele Ghezzi, Hellas Cena, Martina Chiara Pascuzzi, Corrado Regalbuto, Rossella Lamberti, Virginia Rossi, Matteo Manuelli, Alessandra Bosetti, Gian Vincenzo Zuccotti

**Affiliations:** 1 Pediatric and Adolescent Unit, Department of Internal Medicine, University of Pavia, 27100 Pavia, Italy; 2 Pediatric Department, “Vittore Buzzi” Children’s Hospital, 20154 Milan, Italy; Michele.ghezzi@asst-fbf-sacco.it (M.G.); martina.pascuzzi@unimi.it (M.C.P.); rossella.lamberti@unimi.it (R.L.); virginia.rossi@unimi.it (V.R.); alessandra.bosetti@asst-fbf-sacco.it (A.B.); gianvincenzo.zuccotti@unimi.it (G.V.Z.); 3 Department of Health Sciences, University of Milan, 20142 Milan, Italy; 4 Laboratory of Dietetics and Clinical Nutrition, Department of Public Health, Experimental and Forensic Medicine, University of Pavia, 27100 Pavia, Italy; hellas.cena@unipv.it; 5 Clinical Nutrition and Dietetics Service, Unit of Internal Medicine and Endocrinology, ICS Maugeri IRCCS, 27100 Pavia, Italy; m.manuelli88@gmail.com; 6 Pediatric Unit, Department of Maternal and Children’s Health, Fondazione IRCCS Policlinico S. Matteo and University of Pavia, 27100 Pavia, Italy; corrado.regalbuto01@universitadipavia.it; 7 Pediatric Clinical Research Center Romeo ed Enrica Invernizzi, Department of Biomedical and Clinical Science “L. Sacco”, University of Milan, 20157 Milan, Italy

**Keywords:** obesity, asthma, diet, nutrition, nutrients, children, pediatrics

## Abstract

Childhood obesity rates have dramatically risen in numerous countries worldwide. Obesity is likely a factor in increased asthma risk, which is already one of the most widespread chronic respiratory pathologies. The pathogenic mechanism of asthma risk has still not yet been fully elucidated. Moreover, the role of obesity-related inflammation and pulmonary overreaction to environmental triggers, which ultimately result in asthma-like symptoms, and the importance of dietary characteristics is well recognized. Diet is an important adjustable element in the asthma development. Food-specific composition of the diet, in particular fat, sugar, and low-quality nutrients, is likely to promote the chronic inflammatory state seen in asthmatic patients with obesity. An unbalanced diet or supplementation as a way to control asthma more efficiently has been described. A personalized dietary intervention may improve respiratory symptoms and signs and therapeutic response. In this narrative review, we presented and discussed more recent literature on asthma associated with obesity among children, focusing on the risk of asthma among children with obesity, asthma as a result of obesity focusing on the role of adipose tissue as a mediator of systemic and local airway inflammation implicated in asthma regulation, and the impact of nutrition and nutrients in the development and treatment of asthma. Appropriate early nutritional intervention could possibly be critical in preventing and managing asthma associated with obesity among children.

## 1. Introduction

Obesity is a well-known leading public health issue. The World Health Organization (WHO) indicated that by the time the statistics for the year 2016 were compiled, nearly 41 million children under 5 years of age and more than 340 million children and adolescents between the ages of 5 and 19 were either overweight or obese; in the European population, the proportions were 25–70% being overweight and 5–30% with obesity [1]. 

Obesity is likely a factor in increased asthma risk, which is already a serious chronic respiratory ailment representing a public health issue around the world [2,3]. Although asthma affects people of all ages, it disproportionately affects children [4]. In 2016, the Centers for Disease Control and Prevention (CDC) stated that asthma affected approximately 6.5 million children (~9% prevalence) in the US [5], while about 334,000,000 people worldwide were affected.

The mechanisms that lead to pediatric obesity-related asthma are multifactorial and include changes in lung mechanics, dietary intake, systemic inflammation, and metabolic disorders [6].

Asthma tends to be more severe in well-built and/or extremely well-built children and teenagers compared to normal-weight ones and is typically characterized by more frequent exacerbations and lower response to medical therapy [7,8,9,10,11,12,13,14]. Diet is an important adjustable element in the development and progression of asthma. Food-specific composition of the diet, in particular fat, sugar, and low-quality nutrients, is likely to promote the chronic inflammatory state seen in asthmatic patients with obesity [15,16,17]. The use of dietary supplementations or alterations as a means to improve asthma control has been described in several studies. 

This narrative review’s objective is to present and investigate more recent literature on pediatric asthma as a result of obesity, focusing on the risks among children with obesity in developing asthma, the role of adipose tissue as a mediator of systemic and local airway inflammation implicated in asthma regulation, and the impact of nutrition and nutrients in the development and treatment of asthma. Appropriate early nutritional intervention may be crucial for the avoidance and handling of pediatric obesity-associated asthma. 

## 2. Methods

A narrative review was undertaken [18]. The authors, M.C.P., C.R., R.L., V.R., and M.M., independently clarified the most useful research manuscripts submitted up to June 2021 (original papers, clinical trials, metanalysis, and reviews) in English over the previous 15 years. They did not include case reports or series and letters. Manuscripts in the authors’ specific field were combed with a combination of the keywords below: obesity, teenagers, children, asthma, asthma as a result of obesity, pediatric, nutrients, asthma and diet, asthma and nutrients. We conducted searches using the following on-line medical literature databases: PubMed, Scopus, EMBASE, and Web of Science. The inclusions were analyzed and peer reviewed by V.C., E.V., H.C., M.G, M.C.P., C.R., R.L., V.R., and M.M. Our results were examined by the group and the final draft was drawn up.

## 3. Pediatric Obesity and Asthma

Obesity is the result of an elevated proportion of body fat, secondary to both a positive energy balance and weight gain because of several reasons, including the energy intake/output ratio, increased daily consumption, particularly that of energy-dense food, and reduced physical activity due to an increasingly sedentary lifestyle [7,8,19]. This has now become a serious worldwide public health concern in adults and children. From 1975 to 2016, the global age-standardized pediatric obesity occurrence rose from 0.7 to 5.6% and from 0.9 to 7.8% in females and males, respectively [9].

Obesity is a notable risk factor and disease modifier of asthma and very often aggravates its severity. Asthma is the most prevalent chronic pathology in pediatrics [10] and is one of the first 20 serious ailments in disability-adjusted life-years (DALYs) in this group; in middle childhood (6 to 12 years), it represents one of the top 10 causes. Airway hyper-responsiveness, inflammation, and obstruction/remodeling are the main structural and anatomical features.

Many meta-analyses and prospective and cross-sectional research has demonstrated that obesity independently predicts the occurrence of asthma, with a relative risk of 1.2–1.8 for incident asthma in patients with overweight/obesity [11,12,13,14,20,21,22]. 

A study by Camargo et al. shows that a BMI higher than or the same as 30 kg/m^2^ significantly raises the chances of late-onset asthma, with an odds ratio of 2.6 [23]. The European Community Respiratory Health Survey (ECRHS) also discovered an association between asthma and obesity, reporting a higher risk in girls than boys. 

Regarding the risk of developing allergic asthma, Changstang showed that boys who developed obesity in the prepubertal period (9–11 years old) had a twofold increased risk compared to the girls, although this observation was only valid in the patients with consistently high BMIs [24]. Several scientific data suggest that hormonal characteristics in the prepubertal age, pro-inflammatory hormonal factors (e.g., leptin and adiponectin), and estrogen may associate asthma and obesity [24,25]. 

A longitudinal cohort study conducted in Tucson, Arizona, USA showed that a high BMI is linked to developing asthma following the teenage period. Although there have been multiple studies concerning the association between asthma and gender, no unequivocal conclusion has been reached; therefore, further research on this topic is needed [23,24,25,26]. Remarkably, a recent study reports that asthma may result in obesity: in fact, infants suffering from asthma have a higher chance of becoming obese. A number of longitudinal studies have demonstrated that obesity or overweight frequently precedes incident asthma [11,27,28,29,30,31,32,33,34,35,36,37]. As reported in a study involving 108,000 participants [38], a high initial BMI and subsequent weight gain while pregnant were independently linked to a ~15–30% chance of children born to these mothers developing asthma, leading to the possibility that an obesity-induced increase in asthma risk may have its onset in utero [38]. 

The pathogenic mechanism of asthma risk in patients with obesity has not yet been fully elucidated. The role of obesity-related inflammation in the exaggerated pulmonary responses to environmental factors leading to asthma-like symptoms and the mechanical effect and importance of dietary characteristics that might lead to both obesity and asthma are well recognized [39,40,41,42,43,44].

The “asthma” phenotype is typically characterized by the occurrence of additional symptoms, poorer control, the increased frequency and severity of acute episodes, impaired response to inhaled corticosteroids, and a reduced life quality compared to a different phenotype [37,38,39,40,41]. 

Diagnostic and therapeutic management of asthma in pediatric subjects with obesity.

The first step in diagnosing asthma is to collect the patient’s history and conduct a physical examination, performing PFTs, including spirometry and lung volume measurements. Normal spirometry along with normal lung volume in a patient showing symptoms should lead to further testing, including airway hyper-responsiveness, using a 6 min walk test (6MWT) or cardiopulmonary exercise test (CPET), or a methacholine challenge. To evaluate the degree of atopic airway inflammation, fractionated exhaled nitrous oxygen (FeNO) along with indicators of inflammation and metabolic dysregulation can be used as useful tools to understand the etiology of lung dysfunction in obese or overweight children [22,45,46].

Children with excessive weight usually show reduced pulmonary and chest wall compliance, which ultimately contributes to increased respiratory work with oxygen used while exercising, causing the typical superficial and rapid breathing pattern seen in many obese people [22]. Moreover, having identified that obesity-related inflammation may have a fundamental role, EIB in infants with asthma and obesity measured by CPET seems to linearly correlate with leptin and inversely correlate with adiponectin concentration [22,47]. FeNO, which is measured in the exhaled breath of asthmatic patients, is known to correlate with eosinophilic airway inflammation [22,48].

Various studies have reported contradictory results concerning the FeNO significance in the obesity situation with and without asthma. Some obesity-related asthma studies showed low FeNO values or a lack of evidence for any important association between FeNO and the severity of the ailment [37,46,47,48], whereas others showed higher FeNO values in asthmatics linked to the atopic status and not influenced by obesity [49,50,51,52,53,54]. Treatment for obesity-related asthma is obviously not as linear as it is for asthma without obesity; nasally taken corticosteroids and long-acting beta agonists, along with leukotriene inhibitors, are less effective for asthmatic patients with obesity, since they target inflammation driven by eosinophils [55]. Immunotherapy such as omalizumab, reslizumab, mepolizumab, and benralizumab target Th-2 mediated inflammatory pathways [22,56]. Considering this evidence, the lack of atopy in asthmatic patients with obesity suggests that these medications would be less effective [57]. 

As obesity affects several asthma variables, weight loss/a reduction in asthmatic children with excessive weight is a crucial target for contemporary treatment approaches. 

Concerning diet-induced weight loss, as detailed below, a lot of research has explored the function of changing asthmatic obese patients’ diets. It is important to again underline the fact that weight loss at any time in a patient’s life (especially during the pediatric stage, which is the most delicate period) has been associated with improved symptoms [58,59].

## 4. “Obese Asthma” Phenotype

Several scientific studies correlate asthma and obesity in childhood; nevertheless, it has not been clearly defined whether asthma triggers the start of obesity or the opposite is the case. There are many peer reviewed papers which show that patients with obesity have an elevated chance of not only a new onset of asthma [11,12,30] but also for increased intensity of this pathology [41,60], with severe exacerbations and an impaired response to medications. Conversely, some findings support the role of childhood asthma in the onset of obesity [39,40,41].

Considering these contrasting elements, we can say that asthma and obesity may be comorbid, or asthma could lead to obesity, and obesity may confound its diagnosis [61]. However, studies performed in recent years point toward the “obese asthma” phenotype, in which obesity is a transformer element for asthma [62,63], characterized by additional symptoms, worse asthma exacerbations, and a lower response to inhaled corticosteroids [55,64,65].

Diaz [66] categorized two phenotypes, early-onset and late-onset obese asthma, classified by the age it started, gender, airway function (FEV1, FVC), atopic/non-atopic status, airway hyper-reactivity, symptom score, airway inflammation, and Th1–Th2 profile. Early-onset asthma occurs in children under 12 years old with obesity, which irritates underlying asthma; these patients are allergic, and inflammation is predominantly eosinophilic. In the late-onset asthma phenotype, patients are not allergic, and they show more prevalent neutrophilic airway inflammation with a low response to treatment with large doses of inhaled corticosteroids and long-acting bronchodilators.

## 5. Asthma Endotype

Asthma is a diverse ailment with different clinical manifestations (phenotypes) and complex pathophysiological mechanisms (endotypes).

Type 2 asthma is the most commonly found phenotype, with early onset, occurring either with or without any other atopic presentations [67]. The term type 2 immune response refers to the involvement of Th2 lymphocytes, ILC2, immunoglobulin E (IgE)-secreting B lymphocytes, T lymphocytes such as natural killer (NK-T), mast cells, basophils, eosinophils, and their cytokines. Interleukin (IL)-4, IL-5, IL-9, and IL-13 are the most relevant cytokines produced by Th2 cells [68,69]. While IL-4 is involved in the allergen-specific response (synthesis of IgE), IL-5 plays a crucial role in the recruitment and survival of eosinophils [67]. IL-13 is involved in an inflammatory boost in the airway mucosa that predisposes one to asthma exacerbations and remodeling changes.

Eosinophilic non-allergic asthma is mediated by ILC2 production, induced by the release of IL-10, transforming growth factor beta (TGF-β) and alarmins, IL-25, IL-33, and thymic stromal lymphopoietin (TSLP) [67]. Alarmins act as intercellular signals and enhance the immune response by interacting with pattern recognition receptors (PRRs) [68], and their release, mainly in airway epithelial cells, can be triggered by different external agents, pollutants, tobacco smoke, or viruses [67]. Moreover, they can also activate dendritic cells [70] and contribute in different ways to the inflammatory airway response in eosinophilic non-allergic asthma [67]. TSLP, which is overexpressed in patients with severe asthma [71,72], enhances both chemotaxis and eosinophil activation [67]. A co-occurrence of IL-33 serum concentrations and the occurrence of severe asthma has been noted in recent studies [73]. 

Type 2 inflammation is usually responsive to treatment with inhaled corticosteroids.

Most typically found in adults is noneosinophilic asthma, which is characterized by a neutrophilic or paucigranulocytic inflammatory pattern. The predominant cytokines in neutrophilic asthma are IL-17, IL-21, and IL-22, which are generated and secreted by Th1 and Th17 cells.

Neutrophils are the main source of IL-6 generated in the airways of subjects with asthma, and increased levels of IL-6 have been found in asthmatic patients, although the association between IL-6 and severe asthma has only been demonstrated in adults [67,74,75,76]. 

The nucleotide-binding oligomerization domain-like receptor family pyrin domain containing 3 (NLRP3) inflammasome represents another mechanism that drives neutrophilic airway inflammation in the lung. 

This molecular complex triggers the initiation of IL-1β and IL-18 to promote Th17-dependent inflammation [77]. In addition, this is one of the mechanisms underlying chronic inflammation due to obesity activated by saturated fatty acids and cholesterol and oxidative stress via Toll-like receptor 4 [67]. 

A fundamental feature of asthma is airway remodeling due to chronic insult and inflammation, resulting from allergen exposure in sensitized patients and environmental triggers (tobacco, pollution, microbes). All molecules previously mentioned are involved in tissue remodeling, with thickening of the airway walls, increased collagen deposition, and smooth cell hypertrophy. 

In particular, TGF-β stimulates collagen deposition and contributes to airway remodeling, but it additionally takes on an anti-inflammatory role in inhibiting immune system cells (T cells, B cells, Th1, Th2) and interferon (INF)-γ and IL-2 production. Moreover, it converts naïve T cells into Tregs and Th17, promoting immune tolerance.

Besides evidence from the underlying etiopathogenetic mechanisms, the inflammatory pattern characterizing the airway lumen in obesity-related asthma is neutrophil-dominant, rather than eosinophilic. In particular, the high neutrophil count is associated with elevated levels of IL-17A, which in turn is involved in neutrophil chemotaxis. Similarly, neutrophil counts and IL-6 levels were significantly increased in a group of obese adults with severe asthma compared with patients without obesity [67]. 

## 6. Adipose Tissue-Associated Inflammation and Asthma

While in adults, asthma is mostly associated with obesity-related mechanical conditions, in children, the immunomodulatory mechanism is considered predominant. 

When the deposition of excessive adipose tissue occurs, the pathological immune system is activated, provoking a chronic low-grade inflammatory condition called “meta-inflammation” [7,78,79,80], which plays a major part in the association between obesity and its multiple complications, including cardiovascular diseases, diabetes, dyslipidemia, and respiratory sequelae such as asthma [80,81,82]. 

Meta-inflammation is defined as the activation of inflammatory signaling pathways and the recruitment of proinflammatory immune cells, dysregulated cytokine production, and increased acute-phase reactants [83,84,85]. 

A large variety of proinflammatory immune cells inhabit obese adipose tissue, recruited by the proinflammatory cytokines secreted by adipocytes, including activated macrophages, NK cells, mast cells, dendritic cells, B cells, cytotoxic T cells, and Th1 cells. These cells can themselves stimulate adipocytes and lead to a major output of proinflammatory factors, such as TNF alpha, IFN-γ, IL-1β, and IL-6. This particular environment is involved in perpetuating both local and systemic inflammation.

Macrophages are critical in developing this chronic inflammatory state [86]. We can divide macrophages into two main subtypes according to their secreted cytokines: M1, with a proinflammatory role, and M2, with an anti-inflammatory role [87]. The nutritional status causes changes in macrophage polarization in adipose tissue, with the M2 phenotype prevailing in lean children and M1 in those with excessive body weight [17,80]. M1 macrophages are defined by the expression of TNF alpha and inducible nitric oxide synthase, primarily expressed in visceral adipose tissue (VAT) and involved in the inflammatory response. Conversely, M2 macrophages, prevailing in the adipose tissue of normal-weight people, express genes encoding anti-inflammatory cytokines such as IL-10 [88,89]. 

Adipocyte hypertrophy and local hypoxia play major roles in directing this diversification due to adipocyte expansion, which upregulates the secretion of inflammation-related adipokines [90]. In response to adipocyte death, proinflammatory M1 macrophages surround dead and dying cells and remove debris from the damaged area. Nowadays, obesity-related meta-inflammation is thought to be caused by the infiltration of adipose tissue by M1 macrophages, along with their altered function and anatomical localization. 

B and T lymphocytes are adaptive immune cells. CD4+ T lymphocytes can be divided into T helper subsets Th1, Th2, Th17, and Treg, which differ in their surface markers, types of secreted cytokines, and cellular targets [91]. Several studies have observed that in an inflammatory condition, which is obesity-related chronic low-grade inflammation, there is a rise of circulating Th17 and Tregs [80,92,93]. Th17 lymphocytes are relevant in both innate and adaptive immunity. They secrete IL-17, which binds to IL-17 receptors located on innate immune cells, stimulating the production of bioactive molecules (GCS-F and IL-8), which leads to neutrophil recruitment. Th17 also leads to the release of several other proinflammatory cytokines, such as IL-6 [94]. On the other hand, Treg cells are abundant in lean adipose tissue and are involved in the regulation of autoimmunity, allergy, microbial infection, and oncogenesis, playing an important role in the maintenance of tissue homeostasis [88,89]. Treg lymphocytes increase in an inflammatory state, and in adipose tissue, interact with macrophages, preventing metabolic diseases and reducing local inflammation [80]. In addition, the functions of Treg and M1 macrophages are antagonistic; thus, their imbalance is a critical contributory factor in obesity [80,95,96]. Th17/Treg imbalance, with increased Th17 and reduced Treg, can mediate the occurrence of obesity-related inflammation and metabolic disorders [97]. 

Regarding the bioactive molecules of adipose tissue, proinflammatory and anti-inflammatory molecules can be identified [85]. 

Proinflammatory adipokines and chemokines include leptin, resistin, lipocalin 2 and IL-6, TNF alpha, and C-reactive protein (CRP). 

Leptin has pleiotropic characteristics and affects the human body systemically, playing an extensive role in the immune system by increasing the secretion of proinflammatory cytokines by macrophages (TNF alpha, IL-6, and IL-1) and promoting macrophage phagocytosis [98,99,100]. Regarding its role in adaptive immunity, leptin enhances the proliferation of CD4+ T lymphocytes and their orientation into a pro-inflammatory Th1 phenotype by increasing pro-inflammatory cytokines, such as INF-gamma and IL-2, and decreasing the generation of anti-inflammatory Th2 production cytokines, such as IL-10 and IL-4 [17,80,101,102]. It also stimulates the replication of Th17 cells, but reduces the proliferation of Treg cells [17]. Additionally, leptin raises the B lymphocytes proliferation levels [103].

Resistin is an adipokine also referred to as adipose tissue-specific secretory factor. Notably, it is involved in the inflammatory process, for example, inducing the expression of IL-6 and TNF alpha, and is involved in one of the pathways that induce the differentiation of proinflammatory macrophages [80,85,104]. 

Lipocalin 2 (LCN2), a glycoprotein expressed in adipose tissue and the liver, participates in innate immune system reaction, being critical in the acute phase response to infection [79,83,85]. 

Tumor necrosis factor (TNF) alpha, a proinflammatory cytokine produced by macrophages, monocytes, T lymphocytes, and adipose tissue, has high serum concentrations in patients with obesity, and its secretion is exacerbated by leptin. It causes inflammatory changes, especially in the endothelium, triggering vascular dysfunction and hypertension [84,105]. TNF alpha affects glucose and fatty acid metabolism, reducing the secretion of some adipokines, such as adiponectin, and is associated with insulin resistance onset [106,107]. 

IL-6 has elevated levels in obese sufferers’ serum and is involved in reducing adiponectin secretion and increasing CRP production, exacerbating systemic inflammation [84]. Roughly a third of the IL-6 in the bloodstream comes from the adipose tissue and is principally secreted by endothelial cells, macrophages, and fibroblasts [108,109]. In asthma, elevated serum IL-6 has been found to be associated with lower pulmonary function and greater exacerbation risk, independent of obesity [67].

In fact, IL-6 has pleiotropic effects and participates in the polarization of naive T cells into Th17 cells through IL-17 production [110]. IL-17 leads to neutrophil recruitment in the respiratory tract, while IL-6 increases neutrophil activation [67]. Several studies have shown that IL-6 is associated with the most severe clinical phenotypes and corticosteroid resistance; moreover, it is inversely related to the predictive percentage of FEV1 [75]. For these reasons, it is critical in the association involving lung function, asthma severity, and metabolic syndrome [111].

CRP is an acute phase plasma reactant that is mostly secreted by hepatocytes. Various studies suggest a tight link between obesity and high CRP serum levels, which not only contributes to an exacerbation of the systemic inflammatory state, but also suggests that an increase in serum levels of CRP has an inflammatory effect on obesity [85,112,113]. Furthermore, children with larger waist circumferences are shown to have higher CRP levels [113]. 

Anti-inflammatory adipokines and cytokines include apelin, adiponectin, fibroblast growth factor 21 (FGF21), and interleukin 10 (IL-10).

Adiponectin is the main anti-inflammatory and insulin-sensitizing cytokine secreted by healthy adipose tissue; its serum levels are lower in patients with obesity and increase with weight loss [85,114,115,116,117]. In blood vessel walls, adiponectin inhibits the adhesion of monocytes by reducing adhesion molecules and transforms macrophages into foam cells by inhibiting the expression of scavenger receptors [85]. In obese adipose tissue, adiponectin depletion induces a macrophage switch from the anti-inflammatory M2 phenotype to the pro-inflammatory M1 phenotype, which will subsequently attract further peripheral monocytes that will be polarized to M1 through the secretion of proinflammatory cytokines [110,111,112]. 

Apelin is an anti-inflammatory peptide encoded by the APLN gene that is expressed in several organs and tissues, such as adipose tissue, liver, heart, lung, brain, and gastrointestinal tract [118]. This adipokine contributes to glucose metabolism, lipolysis, cell proliferation, and angiogenesis; at higher serum levels, which increase with weight loss, it improves insulin sensitivity [85,88,119]. 

FGF21 is a protein encoded by the FGF21 gene and secreted by the liver, skeletal muscles, and adipose tissue. It is known to have anti-inflammatory properties; in addition, it positively affects lipid and glucose homeostasis, acting directly on white adipocytes, inhibiting lipolysis, and stimulating glucose uptake [85,120]. 

IL-10 is an immunosuppressive cytokine secreted by activated M2 macrophages and Th2 lymphocytes. It antagonizes TNF alpha and IL-6 proinflammatory effects and has anti-inflammatory and endothelial protective properties [84,105]. 

**Figure 1:**
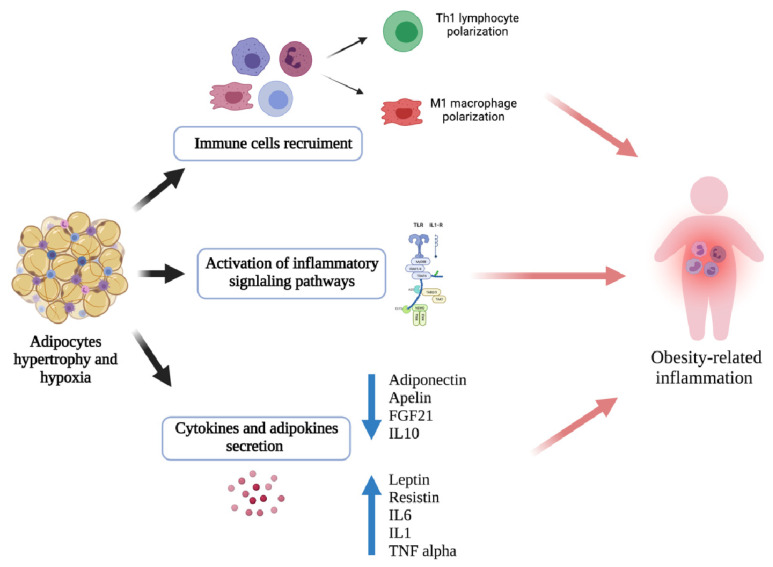
Adipose tissue and obesity-related inflammation.

Adipose tissue releases pro-inflammatory adipokines, which influence the response of the lung to external stimuli, leading to asthma-like symptoms. The role of adipose tissue in the development of obesity-related inflammation is shown in Figure 1. 

## 7. Obesity and Pulmonary Function

Obese patients suffer a diminution in expiratory reserve volume (ERV) and resting lung volume, called functional residual capacity (FRC), because of a tiny increment in intra-abdominal and pleural pressure (ERV) and the lung’s resting volume, which is called functional residual capacity (FRC) [121].

One of the first studies exploring the connection between childhood obesity and lung function was carried out by Lazarus et al., showing that FEV1 levels adjusted for age and forced vital capacity (FVC) were greater in subjects with higher weights [122]. A recent meta-analysis considering studies of children and adults demonstrated that obesity in pediatric patients was related with normal or higher FEV1 and FVC levels [123]. This unexpected increase in these values is ascribed to airway dysanapsis, where the growth of pulmonary tissue is incongruent with the caliber of the airway [124]. 

Obesity has physical and mechanical effects on the airway tract, which is an important connection between childhood obesity and asthma. It is well known that pulmonary function is affected in obese adults with and without asthma. A significant prospective study on children and adult patients diagnosed with asthma showed a considerable decrease in baseline FVC in adults with obesity but not in children [125].

Obesity has various effects on pulmonary mechanics, which can be measured via detailed lung function testing [22].

Pulmonary function deficits, as summarized in a large meta-analysis [123], were first evaluated using spirometry, a common lung function test. 

Since children with obesity have early somatic growth, children with excessive body weight reported a rise in absolute FVC levels, with a larger FEV1 but lower FEV1/FVC ratio, probably ascribable to the disproportionate growth of lung size compared to airway caliber.

The thoracic and abdominal deposition of fat also impact lung volume, but a limited number of studies have quantified lung volume in children [22,126,127,128,129,130,131,132,133,134,135].

FRC and ERV levels seem to be lower in patients with obesity, suggesting the early onset of obesity-mediated pulmonary function deficits [121,122,123]. 

Abdominal fat deposition also leads to low lung volume because of low tidal volume breaths, probably leading to alveolar hypoventilation along with increased airway resistance, which subsequently results in airway hyper-responsiveness, culminating in increased respiratory rates and higher breath effort [22,124,125,126,134]. 

Obviously, not every obese child will develop asthma, yet there is no direct correlation between incident asthma and obesity. Thus, other aspects of obesity, such as metabolic dysregulation, may explain the noted alterations in lung function [22].

These findings suggest a relationship between abdominal fat, asthma, and modified lung mechanics. It is well known that abdominal fat is a risk factor for metabolic dysregulation [136,137].

Overweight asthmatic children typically display a higher prevalence of metabolic syndrome and its related morbidities, such as insulin resistance and dyslipidemia [22]. Insulin resistance has also been correlated to incident asthma, greater asthma severity, and impairments in lung function [132,133,134,135]. 

Some recent findings suggest that insulin seems to be linked with non-atopic systemic immune responses, mediating the association of immune responses with pulmonary function; furthermore, insulin appears to be involved in increased airway smooth muscle contractility [22,138,139,140,141,142]. 

Dyslipidemia also seems to be more prevalent in asthmatic patients [139,143,144]. High levels of cholesterol and its metabolites activate histamine release, promoting the contraction of smooth muscle cells. Increasing fat intake with diet leads to neutrophilic inflammation of the respiratory tract through IL-1b-mediated inflammasome activation [145].

Asthmatic children with excessive body weight present higher maximal oxygen respiration and glycolytic rates than normal-weight asthmatic children, which produces more oxidants. In obese patients, this can be expressed by the decreased nitric oxide (NO) bioavailability, which is an inhibitor of mitochondrial respiration [145]. 

There is mitochondrial dysfunction is both airway epithelial cells of asthmatic patients and adipose tissue [146]. 

Reactive oxygen species (ROS) production and glutathione degradation reduce the damage repair capacity of the respiratory epithelium. The degree of oxidative stress in the airways correlates positively with asthma severity and steroid therapy resistance [147].

**Figure 2:**
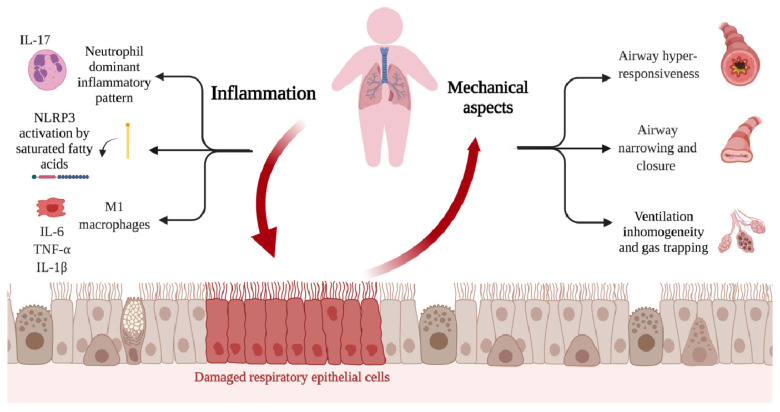
The multifactorial role of obesity in the asthma.

The multifactorial role of obesity in asthma is shown in Figure 2.

## 8. Impact of Nutritional Status on Asthma Prevention and Treatment

Improving the nutritional status of children with asthma helps to mitigate chronic inflammation and reduce the burden of living with a chronic disease. Nutrition early in life and at developmental ages may have an impact on asthma prevention, treatment, and empowerment.

### 8.1. Early-Life Nutrition and Asthma Prevention

#### 8.1.1. Breastfeeding

Breastmilk provides the optimal nutritional intake early in life, influences the gut microbiome, and helps to develop the immune system [148,149]. Vitamin A, immunoglobulins, and growth factors support the integrity and homeostasis of the intestinal mucosal barrier and make breastfeeding crucial in tolerogenic immune response development during early childhood [150,151]. 

It has also been associated with a lower incidence of allergic diseases. Breastfeeding has a protective and dose-dependent impact on preschool wheezing, although the mechanisms are not fully elucidated [152]. Preschool wheezing is commonly triggered by viral respiratory infections, and this finding supports the rationale that breastmilk plays a protective role by reducing the impact of such infections [153]. Systematic reviews and meta-analyses have shown that this protective effect has a tendency to decline in elder infants when disparate elements can affect breathing morbidity [154]. Several studies have also focused on the impact of the timing of breastfeeding. A 2008 American Academy of Pediatrics (AAP) report concluded that sole breastfeeding for at least 3 months is protective against wheezing in the early years of life [155]. A more recent finding indicates that sustained breastfeeding, although not exclusive, is protective against wheezing in the first 2 years of life [155]. The protective role of breastfeeding in the development of asthma is well established. More prolonged breastfeeding also leads to a decreased risk of developing childhood asthma, which, although it decreases over time, remains evident at school age [156]. Although the pathogenesis remains controversial, the WHO and AAP recommend breastfeeding as the first choice of feeding for infants and young children [157,158].

#### 8.1.2. Infant Formula

The early introduction of infant formulas during the first 6 months of life was found to increase the risk of asthma almost twofold at 3 years of age [159,160]. The AAP has proposed the preventive use of a hydrolyzed formula for infants at high risk of atopic disease who cannot be exclusively breastfed [155]. Nevertheless, there is still disagreement about the role of partially and extensively hydrolyzed milk formulas in asthma prevention. A number of previous studies could not successfully prove any noticeable difference between partially and extensively hydrolyzed milks in the prevention of asthma and wheezing [161,162,163]. The German Infant Nutritional Intervention study, which is a prospective randomized double-blind study, provided no results to support the use of hydrolyzed milks (partial hydrolysate, extensive hydrolysate, and extensive casein hydrolysate) for the first 4 months of life in infants at high risk of allergic diseases who could not be breastfed [164]. After long-term follow-up, the authors found no effect on developing asthma [165]. A decreased asthma prevalence was found in a cluster of 11-to-15-year-old children who had received extensively hydrolyzed casein-based milk [166,167]. A recent birth cohort study suggested that partially hydrolyzed milk is not protective on asthma risk until the age of 2 years and is linked to an increased risk of wheezing at 1 year of age in high-risk infants [168]. Ultimately, a recently published Cochrane review indicated that hydrolyzed formula use in the first days of life over solely breastfeeding has no relevant difference in terms of preventing childhood allergies, notably wheezing and asthma. A lack of evidence supports the use of hydrolyzed formula to prevent allergic diseases among infants not exclusively breastfed [169]. 

#### 8.1.3. Cow’s Milk and Soy Milk

The utilization of cow’s milk in a balanced diet is considered normal; however, due to the greater risk of iron deficiency and iron deficiency anemia, it should be avoided during the first year of life [170]. 

Soy beverages have been related to a higher risk of asthma and bronchial hyper-reactivity [171]. In a case-control study, Han et al. reported a positive relation between the consumption of dairy products (plain milk, chocolate milk, cheese, yogurt, tacos, or burritos) and increased pro-inflammatory IL-17F concentrations [172,173]. However, dairy products are fundamental foods rich in nutrients, including protein, calcium, riboflavin, iodine, phosphorus, and vitamin B12. Indeed, there is limited, non-definitive evidence to support the indication to limit the consumption of milk and other dairy products by asthmatic children [174].

#### 8.1.4. Complementary Feeding

It is currently recognized that delaying the introduction of allergenic solid foods is not effective in decreasing the risk of allergic sensitization and atopic disease in children [155,160,175]. A previous study showed no protection against asthma development at 6 years of age with the delayed introduction of solid foods [176,177]. Evidence regarding early complementary feeding and pediatric asthma prevention is contradictory. In a prospective cohort study involving solid foods before the age of 6 months, the risk of asthma at the age of 5 years dropped [178]. The initial assumption that the introduction of oats, fruits, vegetables, and fish before 1 year of age was characterized by a decreased occurrence of wheezing and asthma in infancy [179,180] was subsequently refuted [181]. Special attention was given to the timing of fish introduction for the primary prevention of asthma in view of the high levels of n–3 LCPUFA. Although an earlier introduction of fish (during the second semester of life) has been associated with a decreased risk of allergic sensitization, the protection on the development of childhood wheezing and asthma is still controversial and there are no clear answers [182,183,184]. Recently, the European Society for Pediatric Gastroenterology Hepatology and Nutrition recommended that at any time after 4 months, likely allergenic foods could be introduced when complementary feeding is started [170].

### 8.2. Nutrition at Developmental Age: Prevention, Treatment, and Empowerment

#### 8.2.1. Cow’s Milk

Although as previously stated, unpasteurized cow’s milk is not normally advised because of the danger of potential pathogenic bacterial contamination, many studies have concluded that it may provide protection against allergies and asthma [185]. There has been evidence that during the first years of life, unpasteurized cows’ milk is a contributory factor to asthma, but this effect is reduced by heating the milk [186]. Higher-fat unpasteurized milk, along with butter and n-3 polyunsaturated fatty acids (PUFAs), adds to this result [187]. 

Studies on animals have indicated that unpasteurized and unheated milk stops airway hyper-reactivity and lessens the immune response after exposure to allergens [188]. There may also be an improvement in the barrier function of the intestine, the modulation of the composition of the intestinal microbiota, and the influence of the production of short-chain fatty acids due to milk proteins [189]. Still under debate are the epigenetic mechanisms of action of unpasteurized milk components (PUFA, bacteria, proteins, and vitamins) [190]. 

Currently, however, it is impossible to recommend this milk for the prevention of asthma due to the risk of serious infection after consuming the unpasteurized variety. 

Furthermore, considering the relationship between asthma and food allergy in atopic patients, other studies showed that sensitization to cow’s milk or eggs is a risk factor for the development of bronchial hyper-responsiveness [191]. In fact, a recent study evaluated the beneficial effect of cow’s milk avoidance in terms of the improvement in symptom control in patients with uncontrolled asthma by standard treatment [187]. 

#### 8.2.2. Animal Proteins

Dietary proteins constitute critical macronutrients, providing essential and nonessential amino acids necessary to the healthy development of children [192]. 

The European Food Safety Authority (EFSA) defined the protein requirements for all age groups. The EFSA recommendations for protein are expressed as the Population Reference Intake (PRI) in grams of protein/kilogram body weight/day (g/kg/day), ranging from 0.85 to 1.14 [193]. 

Data collected from a systematic review about protein intake from 0 to 18 years showed that high protein intake in early childhood is associated with a higher BMI with a risk of overweight/obesity later in life, especially when the percentage of energy introduced with proteins at 12 months is more than 15% [194]. An average protein intake of 15% at 12 months of life is therefore recommended. Moreover, the excessive consumption of cow’s milk is associated with a high intake of calories, proteins, and fats and low iron content [170].

Proteins, especially derived from cow’s milk, stimulate the secretion of insulin-releasing amino acids and insulin-like growth factor 1 (IGF-1), facilitating growth, adipogenic activity, and adipocyte differentiation [195]. For these reasons, it is useful to ensure the correct intake of proteins with healthy dietary patterns, preventing the development of non-communicable disease associated with worse asthma symptoms/control. 

Animal proteins (such as red meat) contain n-6 PUFAs, while plant-based proteins contain n-3 PUFAs with anti-inflammatory activity, supporting the positive effects of diets with an higher plant-based protein intake in asthma control [196]. An anti-inflammatory diet should also consider the cattle’s farming practices: organically produced dairy and meats contain higher levels of anti-inflammatory n-3 PUFA. Moreover, when meat is cooked at high temperature, charring heterocyclic amines and polycyclic aromatic hydrocarbons are produced, creating pro-inflammatory products [196]. 

#### 8.2.3. Dietary Patterns

A healthy diet requires that nutrients are consumed in the right proportions to ensure the correct nutritional and energetic supply to the body. Carbohydrates, proteins, and fats are the building blocks for cellular metabolism, while vitamins are required for normal cellular growth, development, and integrity. A healthy diet consists of health-promoting foods, including fresh fruits and vegetables, antioxidants, nuts, and seafood rich in omega-3 fatty acids, while an unhealthy diet consists of saturated and trans fats, animal-derived proteins, and added sugar [197,198].

The intake of nutrients in natural foods seems to have beneficial effects on human health in terms of joint and synergistic action compared to the intake of the same nutrients as supplements and fortification. Even if dietary supplement intake can compensate for the inadequate intake of most nutrients, several studies do not encourage the employment of vitamin, mineral, and fish oil supplements to decrease the risk of non-communicable diseases, including asthma [199]. 

**Figure 3:**
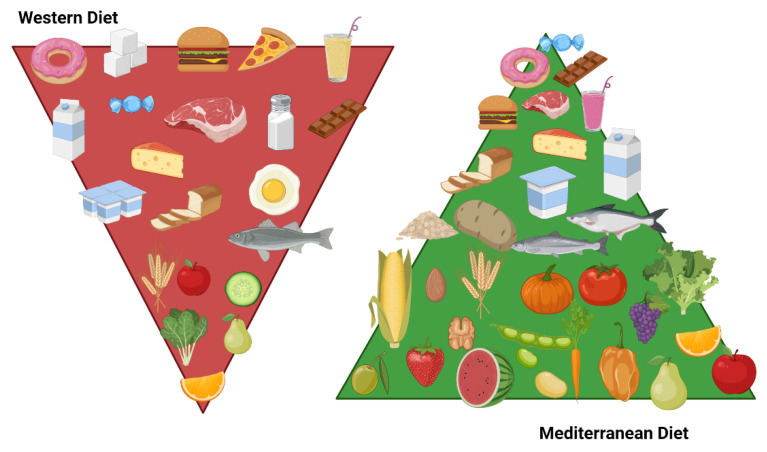
Mediterranean diet and Western diet.

Features of the MD and WD are described in Figure 3.

##### Mediterranean Diet

The key elements of the Mediterranean diet (MD) are daily carbohydrate intake, especially whole grains; fresh and non-processed food consumption; the moderate intake of milk and dairy products, preferably yogurt and low-fat cheeses; the moderate intake of red meat; the preferential intake of fish and moderate intake of eggs; the preferential intake of fruits, vegetables, legumes, and nuts; the use of olive oil as a source of fat; the sporadic intake of sweets, soft drinks, and packaged foods; and water as the main source of hydration. 

The MD is rich in antioxidant, monounsaturated, and n-3 polyunsaturated fatty acids (PUFAs), which mainly derive from olive oil and fish [200,201]. 

The quantity and frequency of food consumption are represented as a pyramid: foods to be consumed in greater quantities are positioned at the bottom, the largest part of the pyramid, while those to be avoided are at the top. In addition, it is fundamental to engage in daily physical activity and avoid sedentary habits [202,203]. 

Evidence for the beneficial role of the MD during pregnancy are inconclusive. Maternal adherence to this diet and particularly olive oil consumption were significantly associated with reduced wheezing in children [204]. Nevertheless, other studies did not find any association with the development of wheezing in the first 15 months of life and at 3 and 4 years of age [205,206], and no association with the development of asthma was found [207,208]. Further investigations are required to better elucidate all the effects of maternal adherence to the MD and its protective effects on asthma. 

Micronutrient dosages as measured in maternal blood during pregnancy or in experimental animal studies could be useful to investigate the impact of the MD during the prenatal period and childhood in developing asthma and allergies in children. On the other hand, transport mechanisms have already been recognized for antioxidants and long-chain PUFAs (fundamental elements of the MD) carried from the maternal to the fetal circulation through the placenta [201]. It is possible that the benefits of the MD are greatest before the allergic response is established, during the stages of early immune development, giving the exposure time window a relevant role [209].

Conflicting studies have investigated the association between the consumption of a Mediterranean diet and the development of asthma during childhood. In general, a fruit- and vegetable-rich diet provides protection due to the positive impacts on inflammation, oxidation, and microbial composition through cytokine release mediation, oxidative stress and redox balance, and immune response [16]. It decreases airway hyper-responsiveness via reduced cytokine production. In fact, fruit and vegetable consumption by patients with asthma is inversely associated with airway neutrophils [210,211]. Consuming fruits and vegetables once a day was associated with reduced incidence of wheezing and asthma [212,213]. A high association with reduced risk of asthma has been noted for oranges [214]. However, some studies have refuted these observations [215]. 

Vegetable consumption is inversely related to asthma prevalence. However, Iikura et al. [216] suggested that flavonoids’ anti-inflammatory action in vegetables is lost upon heating, which might explain the correlation between raw vegetable consumption and well-controlled asthma [16]. A systematic review by the European Academy of Allergy and Clinical Immunology found that increasing fruits and vegetables to reduce the risk of childhood asthma is supported by the current literature [217,218]. The 2015–2020 Dietary Guidelines for Americans advise Americans to eat more fruits and vegetables to lower their risk of developing chronic diseases [16]. 

In addition, current evidence shows the protective effect of the MD on asthma symptoms and exacerbations, infections, hospital admissions, and medication use [16,197,198,199]. In particular, dietary fiber intake is positively related to improvements in lung function (FEV1, FVC, and FEV1/FVC ratio) [212,219,220,221,222,223]. Moreover, fiber influences the gut microbiome, producing metabolites that modulate immune and metabolic responses. Gut microbes (Bifidobacterium and Lactobacillus species) produce short-chain fatty acids (acetate, propionate, and butyrate) through dietary fiber fermentation, leading to decreased inflammatory cytokine expression [224,225]. Dietary fiber reduces blood glucose and increases plasma adiponectin, which has an anti-inflammatory role [226,227]. Alimentary fiber enhances the thickness of the intestinal barrier, preventing proteins from entering the bloodstream and triggering an immune response [228]. For all of these reasons, a model diet that includes five servings of vegetables and two servings of fruits daily should be proposed for its beneficial effects [229]. 

Further, dietary fat intake influences airway inflammation. While saturated fats promote inflammation through the activation of pro-inflammatory NFkB and cyclooxygenase-2 expression mediated by Toll-like receptor-4, unsaturated fats might play a protective role in inhibiting this pathway [224].

Indeed, the MD is an example of a healthy and scientifically accepted lifestyle choice that can protect against major chronic and inflammatory diseases, including asthma [230]. 

##### Western Diet

The Western diet (WD) typically emphasizes the intake of animal products at the expense of fruits, vegetables, legumes, and cereals. This dietary pattern has led to a higher risk of asthma in children [231,232]. The WD in pregnancy has been positively associated with early wheezing risk and is not predictive of asthma onset. In school-age children, a high-fat, low-fiber diet is linked to an augmented risk of wheezing and asthma [158]. A recent study in the Qatari population argued that a high intake of energy-dense foods including desserts, fast food, and soft drinks was likely related to asthma as compared to a diet rich in vegetables, grains, fish, and lean meat [233]. Further studies assessed that a higher intake of take-away products was associated with increased bronchial hyper-responsiveness and that the frequent consumption of hamburgers in particular increased the risk of asthma symptoms. A particular finding is that these associations are independent of obesity-related factors such as BMI [234]. It has also been proposed that the consumption of fast food in childhood may diminish the protection of breastfeeding on asthma risk [235]. 

The WD has led to worsened lung function in asthmatic children [231]. High fat intake is correlated with airway eosinophilia and impaired spirometry results [236]. Additionally, saturated fat consumption is correlated with a higher percentage of eosinophils in sputum [237]. Enhanced airway hyper-responsiveness through increased cytokine production in the lung was also found [238]. In addition, this dietary pattern reduces host antioxidant defenses, increasing susceptibility to oxidative damage [224]. 

As recommended by updated dietary guidelines, daily saturated fat intake should be <10% of one’s total energy intake [16]. 

Therefore, it is desirable to discourage WD patterns in favor of the Mediterranean diet for development and therapeutic support in controlling asthma symptoms [16,202,203,204,205,206,207,208,209,210,211,212,213,214,215,216,217].

**Figure 4:**
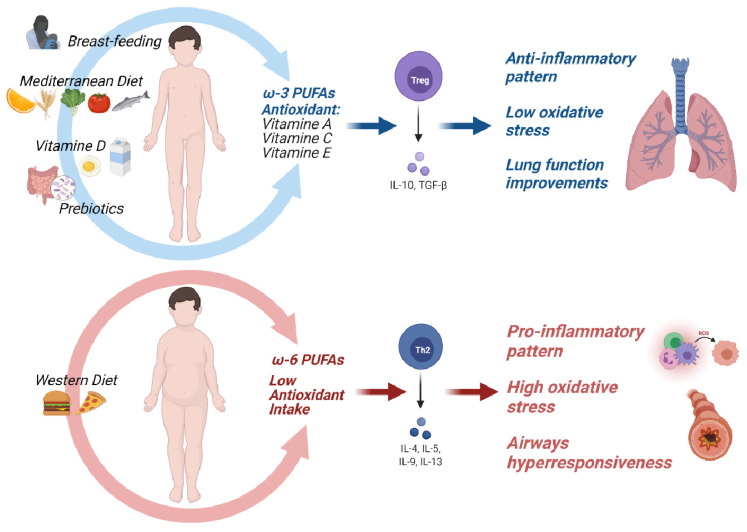
Factors implicated in asthma prevention and treatment.

Factors implicated in asthma prevention and treatment are described in Figure 4. 

### 8.3. Food Supplements

#### 8.3.1. Polyunsaturated Fatty Acids

Fish is the primary dietary source of long-chain polyunsaturated fatty acids (PUFAs), docosahexaenoic acid (DHA), eicosapentaenoic acid (EPA), and docosapentaenoic acid (DPA) [239]. In addition, n-6 PUFAs are typically obtained from animal fats and plant oils, while n-3 PUFAs can be obtained from walnuts [240], leafy greens, and flaxseeds. Linoleic acid (n-6 PUFA) is the predominant fatty acid in the WD with pro-inflammatory activity, while alpha-linolenic acid (n-3 PUFA) is mostly found in the MD. Alpha-linolenic acid (n-3 PUFA) is converted to EPA, which can inhibit arachidonic acid metabolism and interrupt the inflammatory cascade [16]. Otherwise, n-6 PUFA is converted to arachidonic acid, a precursor to PGE2 and leukotriene B4, promoting type 2 T-helper (TH2) cell polarization, neutrophil activation, and IL-6 production. PUFA derivatives can also reduce the accumulation of neutrophils at inflammatory sites [241]. 

In obesity sufferers, adipose tissue releases PUFAs such as oleic and linoleic acids that interact with the free fatty acid receptor 1 and 4 (FFAR1 and FFAR4). FFAR1 regulates insulin secretion, while FFAR4 mediates the secretion of glucagon-like peptide-1, the adipocyte differentiation, and plays an anti-inflammatory effect. It has been shown that activation of FFAR1 and FFAR4 elicits transient increases in [Ca2+] in smooth muscle cells via the classical G pathway, yet FFAR1 is the only receptor for airway smooth muscle contraction. In the lungs, FFRA1 links to n-6 PUFA and induces airway smooth muscle cell contraction and proliferation involved in airway remodeling and hyperresponsiveness, through two signaling pathways, MEK/ERK or PI3K/Akt [242,243,244,245,246] Therefore, the presence of FFAR1 on airway smooth muscle could contribute to the cellular proliferative response to plasma FFAs and could be an important regulator of airway remodeling, especially in obese individuals, playing a key role in linking obesity to asthma. On the contrary, FFRA4 links to n-3 PUFA, exerting anti-inflammatory effects [242]. In fact, a direct selective agonist of FFRA4, TUG-891, was observed to not induce actin reorganization in airway smooth cells, nor proliferation. These observations suggest that FFAR4 does not contribute to the processes previously mentioned [242,243,244,245,246].

The role of fish oil supplementation in the primary prevention of asthma remains uncertain. During pregnancy, fish oil is able to reduce metabolites derived from n-6 PUFA, associated with proinflammatory responses, in favor of omega-3 PUFA metabolites, associated with anti-inflammatory responses. It may also provide epigenetic changes that alter the methylation of specific genes and the acetylation of histones in unborn offspring [221,222,223]. Observational and interventional studies have shown that PUFA levels during pregnancy are inversely proportional to the prevalence of lower respiratory tract infections, persistent wheezing, and childhood asthma [247]. An RCT study suggested that supplementation with DHA is more beneficial in compensating for deficiencies, suggesting the value of identifying the appropriate groups of pregnant women [248]. No association with improved lung function of the unborn child was found [249]. Recently, a Cochrane review argued that the evidence supporting PUFA integration in women when pregnant and while breastfeeding for the primary prevention of allergies in children is scarce [250]. As reported, PUFA supplementation during pregnancy is not associated with a significant protective effect on wheezing and asthma in offspring [251]. Fish intake during pregnancy was not linked to a reduced risk of asthma in the progeny, despite being related to a lower risk of wheezing, eczema, and food allergies in children [252]. 

Observational studies investigating fish intake during childhood have reported conflicting results on its protective role in asthma. The duration of breastfeeding is important in improving the level of n-3 PUFA in infants [253]. The initial beneficial effects of high dietary fish intake on bronchial hyper-responsiveness, upper respiratory infections, and childhood asthma [254,255] have not been confirmed by subsequent studies [241]. One randomized trial showed that the administration of n-3 PUFA was related to a lower risk of recurrent wheezing, but not asthma, at 6 months of age [184,256]. Another RCT study demonstrated a lower occurrence of wheezing, nocturnal cough, and bronchodilator use at 18 months, but highlighted that n-3 PUFA did not prevent asthma at 5 years of age [257,258].

Lee et al. [259] investigated the joint effect of consuming multiple beneficial dietary components on asthma treatment. In this study, 192 asthmatic children aged 10–12 years were recruited from elementary schools in metropolitan Taipei and randomly assigned to the intervention group (fruit plus vegetable concentrate, fish oil, and probiotic supplementation) or the control group (placebo) [259]. The intervention group showed significant improvement in pulmonary function parameters (FVC, FEV1, and FEV1/FVC ratio) and had a significant reduction in short-acting inhaled bronchodilator and inhaled corticosteroid use [239]. Evidence shows that DHA intake can reduce bronchial hyper-responsiveness and eosinophilic airway inflammation, as well as the number of inflammatory cells in bronchoalveolar lavage [260]. Nevertheless, recent trials show no differences in the effect on symptom control between fish oil supplementation and a placebo [239].

Therefore, further studies are needed to clarify the role of PUFA supplementation in the early years of life to prevent and treat pediatric asthma.

#### 8.3.2. Antioxidants

Alimentary antioxidants include vitamins E, C, and A, β-carotene, and flavonoids [16,261].

Vitamin C (ascorbic acid) is a water-soluble antioxidant found in various fruits and vegetables (lemon, orange, pepper, broccoli, spinach, radicchio, and tomato) [262]. The role of vitamin C in asthma prevention may be due to its antioxidant potential and anti-inflammatory properties [263]. Vitamin C is involved in the hydration of airway surfaces and the regeneration of oxidized vitamin E. It also prevents the synthesis of prostaglandin E2 (PGE2), modulates the release of arachidonic acid, neutralizes free radicals, and improves the function of the cells of the immune system [262]. 

Carotenoids and retinol are the two major dietary sources of vitamin A. Orange-yellow fruits and vegetables are rich in carotenoids, along with whole milk, liver, and eggs [262]. Low vitamin A dietary intake is associated with an higher risk of developing asthma and greater severity of the disease [264]. The potential anti-asthmatic effect of vitamin A can be linked to its antioxidant and immune effects [265]. Vitamin A is also fundamental to lung development and the differentiation of lung epithelial cells [266]. Vitamin A supplementation in pregnancy and early life has been observed to improve lung function in offspring [267]. However, again, no reductions in the risk of asthma and no improvements in spirometry parameters were observed in later life [262,266].

Food sources of vitamin E include nuts, seeds, green vegetables, and vegetable oils [268]. The role of vitamin E in asthma prevention may be due to reduced oxidative stress, decreased production of immunoglobulin E, and reduced Th2-mediated response [269,270]. Vitamin E inhibits lipid peroxidation by reducing cell membrane damage and scavenges reactive nitrogen species associated with neutrophilic inflammation [268]. Data regarding vitamin E during pregnancy are controversial. Low vitamin E intake during pregnancy was reported to increase the risk of asthma and wheezing in children for the first five years of life [270]. Higher levels of vitamin E, particularly its alpha-tocopherol isoform, in postpartum maternal plasma concentrations were also associated with a lower likelihood of wheezing in offspring at 2 years of age but not the prevention of asthma [271]. Serum vitamin E concentrations in the first year of life were not correlated with the subsequent risk of developing childhood asthma [272]. 

Flavonoids work like antioxidants and metal chelators, such as iron ions. They also present anti-inflammatory and anti-allergic activities [16]. 

Selenium is a co-factor for the antioxidant enzyme glutathione peroxidase, which is involved in lipid peroxidation prevention. Recent studies have reported that patients with asthma have lower selenium concentrations than people without asthma [16]. 

Antioxidant supplementation is still a debated issue in the treatment of asthma. Some trials show that 1 g/day of vitamin C seems to be protective against airway hyper-responsiveness, leading to less severe and less frequent exacerbations of asthma [167,224].

Patel et al. [273] found that high citrus intake (>46.3 g/d) was associated with a reduced risk of symptomatic asthma, in agreement with others who attributed the same protective effect to apples tomatoes, carrots, and leafy vegetables [214,274].

Pearson et al. [275] reported no benefit from 6 weeks of 500 mg/day vitamin E supplementation, while combinations of β-carotene, vitamin C, and vitamin E were more protective against bronchoconstriction.

In a randomized trial on selenium supplementation by Shaheen et al. [276], participants were divided to receive either a high-selenium yeast preparation (100 µg daily) or a placebo (yeast only) for 24 weeks. Selenium supplementation was not associated with changes in lung function, asthma symptom scores, peak flow, or bronchodilator usage.

In 2014, a Cochrane review tried to assess the effect of vitamin C and E supplementation on health-related quality of life and on exacerbations in adults and children with chronic asthma and subjects without a proper diagnosis of asthma experiencing asthma-like symptoms when exercising. The authors analyzed only one study that included pediatrics (n = 160 children), but could not draw any conclusion due to the lack of specific outcomes in the available trials [277]. 

The poor efficacy of these trials results from the use of individual nutrients rather than their associations in natural food. Regardless, dietary intake of or supplementation with vitamin C, vitamin E, and carotenoids constitutes a reasonable strategy to ameliorate their antioxidant function [224] in asthmatic patients. The consumption of fruits and vegetables should be supported, especially in the youngest of children [239].

In Table 1, current evidence and future perspectives on antioxidants with beneficial effects on asthma are summarized.

#### 8.3.3. Vitamin D

The role of vitamin D in innate and adaptive immunity suggests that it may influence susceptibility to childhood asthma [278,279]. The effect of vitamin D extends beyond the calcium metabolism, and is involved in both innate and adaptive immunity [16,280]. 

Low levels of vitamin D are associated with increased type 2 mediated responses, interleukin-10 production, and reduced T-regulatory cells [278]. Studies have shown that low levels of vitamin D in early life are associated with the onset and persistence of asthma [254,255,256]. Low levels of vitamin D in cord blood and pregnant mothers have been shown to indicate an increased risk of childhood respiratory infections, wheezing, and asthma [281,282,283,284]. In addition, vitamin D deficiency is associated with an increased risk of early allergic sensitization and susceptibility to respiratory infections, risk factors for asthma in preschool and school-age children [283,285].

The role of early vitamin D supplementation in the development of asthma and wheezing is still a subject of study. In the Vitamin D Antenatal Asthma Reduction Trial (VDAART), vitamin D supplementation during pregnancy reduced the incidence of asthma and wheezing in children at the age of 3 years [286]. Furthermore, follow-up of the VDAART cohort showed no impact on the development of recurrent asthma and wheezing in children up to 6 years of age [287]. Overall, vitamin D supplementation in pregnancy and early life may be useful in reducing susceptibility to viral wheezing diseases in preschoolers [158,288,289]. However, there are insufficient data to determine whether postnatal vitamin D supplementation can help in the primary prevention of persistent asthma in school-age children [157,290]. Indeed, adequate intervention studies with long-term follow-up would be needed to recommend vitamin D for use in the primary prevention of wheezing and pediatric asthma. 

Vitamin D status may also affect the severity of asthma through airway remodeling. It plays a pivotal role in directly inhibiting airway smooth muscle cell growth and contractility and fibroblast proliferation [291,292]. Indeed, vitamin D serum concentrations are inversely related to childhood asthma control and a higher risk of hospitalization or emergency evaluation [293,294] and are directly linked to lung function markers, including FEV1 and FVC [291]. However, the association between vitamin D insufficiency and total immunoglobulin E is controversial [292]. 

Vitamin D deficiency is also increasingly prevalent in obese individuals, even if the mechanisms explaining this association are still controversial. Interestingly, vitamin D should play a protective effect in reducing systemic inflammation (lower expression of resistin, IL-6 and TNF-a) [295]. 

Vitamin D supplementation could be primarily considered in obese children with asthma, as both obesity and asthma are characterized by an inflammatory condition and reduced serum 25(OH)D levels [296]. Brehm et al. [297] reported that asthma control is optimized at a serum 25(OH)D level ≥ 40 ng/mL. Although a meta-analysis [298] of vitamin D supplementation trials in pediatric age found that supplementation may reduce the risk of asthma exacerbations, further studies targeted to children with obesity-related asthma should be performed.

Several studies showed that obese children do not have the same response to vitamin D supplementation as normal-weight children. There is no agreement on the recommended dose in this population, although most authors suggest a higher dose than the usual one [299,300]. 

Epidemiological studies have demonstrated that obese children and adolescents are at risk of vitamin D deficiency, due to the deposition of vitamin D in adipose tissue with the decrease in its serum levels. It is not yet known whether vitamin D deficiency could exacerbate the metabolic profile of obese individuals. Considering the inconsistent results and the small number of studies, vitamin D administration is not indicated to improve obesity-related complications [301].

In conclusion, preventing deficiency and insufficiency is preferable to treatment. Supplementation regimes should include 4000 IU of vitamin D daily over a 6-month period at least. In the case of deficiency, weekly boluses of 50,000 IU of vitamin D2 for 6 weeks followed by 1000 IU of vitamin D3 daily are suggested, with monitoring of serum calcium levels [296]. Unsurprisingly, there is growing interest in the potential role of vitamin D in asthma prevention and treatment.

### 8.4. Probiotics, Prebiotics, and Synbiotics: Prevention and Treatment

The gut microbiome in early life is a crucial player in the risk of asthma in children [302]. The so-called gut–lung definition can explain how the gut microbiome might influence lung disease [303]. Specific attention has been given to the mode of delivery and the bacterial composition of the gut in the first month of life, which influence the risk of asthma at 6 years of age [304]. Thus, several studies have evaluated the gut microbiome as a means of asthma prevention. 

Probiotics are live microorganisms. They modulate the composition of intestinal microbiota and can induce an immunomodulating effect, enhancing Th1-mediated responses rather than Th2-mediated ones. Prebiotics are substrates, such as inulin, fructo-oligosaccharides, and galacto-oligosaccharides, used by the host’s microbiota with a beneficial effect. Synbiotics include live microorganisms and energetic substrates that can have a beneficial effect on the body [305,306,307].

Several studies on animals have shown that the administration of probiotics induces a reduction in the inflammatory infiltrate and eosinophil count in the airways and prevents the development of airway hyper-reactivity [273,274]. On the other hand, the scientific evidence in humans is not so clear [308].

Oral supplementation with probiotics during pregnancy or early stages of life has been shown to be effective in the prevention of eczema; however, not in the prevention of wheezing and childhood asthma. Another study, though, showed that this type of oral supplementation, performed during the same period previously mentioned, was able to decrease the incidence of wheezing in a subgroup of children with atopic disease [309].

Randomized trials have shown that prebiotics decrease the risk of wheezing and asthma compared with a control group, but due to the small number of participants and events, these results should be interpreted with caution [310]. In a 2-year follow-up RCT, infants at risk of atopy who received prebiotic-containing formula showed a lower incidence of recurrent wheezing than the placebo group [311]. A 2013 Cochrane review reported no significant effect of oral prebiotics on preventing childhood asthma [312]. A recent meta-analysis of RCTs showed that the role of prebiotics in allergy prevention is still uncertain [310]. 

The role of synbiotics in the prevention of allergic manifestations remains controversial. One study reported that the wheezing prevalence was lower in infants with atopic dermatitis fed with extensively hydrolyzed protein formula associated with synbiotics than those randomized to formula without synbiotics [313].

The role of probiotics as a treatment for asthma has been widely studied. A systematic review reported no curative effects on asthma [314]. A subsequent meta-analysis showed no improvement in quality of life, but a reduction in asthma attacks [315]. A recent experimental study showed that probiotics promote the Th1-mediated response rather than the Th2-mediated response [273,285,305].

No study has investigated the curative effect of prebiotics on asthma [305].

As reported by Hassanzad et al. [316], no statistically significant clinical outcomes were observed after synbiotics were added to asthma management in a population of Iranian children younger than 12 years.

At present, the use of probiotics, prebiotics, and synbiotics in the treatment of asthma is not supported by evidence, and further studies are needed to clarify the beneficial effects and make a definitive recommendation.

Table 2 and Table 3 show a summary of age-related dietary effects concerning asthma prevention and treatment.

## 9. Conclusions

Obesity and asthma are two extremely prevalent diseases in children worldwide that are correlated. Even though the underlying mechanism has not yet been fully elucidated, early dietary intervention, as a modifiable and supportive factor in the prevention and management of asthma associated with pediatric obesity, is well recognized [76,149,150,151,152,153,155,156,157,158,159,165,175,176,178,186,187,188,203,210,212,213,214,317,318]. Novel prevention and treatment approaches are attained because of the heterogeneity of asthma pathogenesis.

Both prenatal and postnatal nutrition can influence the immune system at a critical stage of its development and prevent allergic diseases.

Particularly, the protective effect of breastfeeding for the child is clearly established in the development of asthma [149,150,151,152,153,154,155,156,157].

Concerning diet, [16,209,211,212,213,216,217,219,220,221,222,227,228,229,230,231], the Western diet, which is identified by the exaggerated intake of saturated fatty acids and low intake of antioxidants, causes the development of pro-inflammatory molecular mechanisms involved in the persistence of airway inflammation, heightened asthma symptoms, and the deterioration of respiratory function [159,233,234,235,236,237,238,262]. On the other hand, the MD, identified by higher levels of fruit, vegetable, grain, and unsaturated fatty acid intake, is shown to constrict inflammation and the severity of the symptoms and reduce the use of medication. Consequently, promoting the Mediterranean diet while discouraging the WD is advised [16,209,211,212,214,216,217,219,220,221,222,227,228,229,230,231,239,261]. Moreover, the Mediterranean dietary pattern can ensure an adequate antioxidant intake [201].

Vitamin D’s role in innate and adaptive immunity shows that it may have an influence on the susceptibility to childhood asthma. Recently, a meta-analysis found that vitamin D supplementation is likely to reduce the risk of asthma exacerbations [279,281,282,284,285,286,287,288,290,291,292,293,294,295,296,297,298,302]; however, at the moment, there is not sufficient data to determine whether its supplementation may help in the prevention of asthma. 

Lastly, the roles of probiotics, prebiotics, and synbiotics appear to reduce the respiratory inflammatory pattern via the gut–lung axis, but their role in the prevention and treatment of asthma is questionable due to the connection between the gut and lung microbiomes [303,304,305,306,307,308,309,310,311,312,313,314,315,316,319]. In order to describe the role of dietary supplements (such as fish oil, vitamins, probiotics, prebiotics, and synbiotics) in preventing and improving asthma management in children, further studies are necessary.

Personalized dietary interventions may improve respiratory symptoms and signs and the therapeutic response in pediatric obesity-related asthma. Certainly, weight loss/reduction in asthmatic children with excessive weight remains a crucial target for contemporary treatment approaches.

## Figures and Tables

**Table 1:** Summary of current evidence and future perspectives on antioxidants with beneficial effect on asthma.

Nutrient	Dietary Source	Mechanism of Action	Effect on Asthma	References	Future Prospective	References
**Vitamin C**	Lemon, orange, pepper, broccoli, spinach, radicchio, tomato	Prevents the synthesis of PGE2. Modulates the release of arachidonic acid. Neutralizes free radicals and improves immune system cell function.	Antioxidant and anti-inflammatory effects	[241,242]	Regular supplementation with vitamin C to reduce asthma frequency and severity.	[205]
**Vitamin A**	Orange-yellow fruits and vegetables, milk, eggs	Essential for lung development and differentiation of lung epithelial cells.	Antioxidant and anti-inflammatory effects	[244,245]	There are no trials on vitamin A supplementation to prevent or manage asthma.	
**Vitamin E**	Green vegetables, nuts, seeds, vegetable oils	Decreases oxidative stress Decreases IgE production Decreases Th2-mediated response	Antioxidant and anti-inflammatory effects	[247,248]	Administration of vitamin E combined with β-carotene and vitamin C as a prevention for bronchoconstriction.	[254]
**Flavonoids**	Apples, grapes, eomatoes, salad, cabbage	Suppression of superoxide anion activity Iron-chelating action with the prevention of subsequent oxidative damage.	Antioxidant and anti-inflammatory effects	[16]	The anti-inflammatory effect of flavonoids in vegetables decreases with heating, so the consumption of raw vegetables is recommended to control asthma symptoms.	[16,198]

**Table 2:** Early-life nutritional effects for the prevention of asthma overview.

Early-Life Nutrition		
Type	Prevention	
	Results	References
**Breastfeeding**	Protection with a dose-dependent role on preschool wheezing and asthma.	[152,153,154,155,156]
**Infant Formula**	No evidence to show that the use of hydrolyzed formula versus conventional milk in the prevention of wheezing and asthma.	[161,162,165,166,168,169]
**Soy Milk**	No evidence to show it offers any protection against asthma.	[171,172,174,190]
**Timing of Complementary Feeding**	No evidence to show it offers any protection against asthma.	[182,183]

**Table 3:** Developmental-age nutrition effects on the prevention and treatment of asthma overview.

Developmental Age Nutrition				
Type	Prevention		Treatment	
	Results	References	Results	References
**Cow’s Milk**	No effective role in preventing asthma.	[171,172,174,190]	No effective role in treating asthma.	[171,172,174,190]
**Dietary Patterns**				
**Mediterranean Diet (MD)**	Is shown to protect against preschool wheezing and asthma.	[16,156,212,216,217,219,220]	Is shown to protect against asthma exacerbations and lung function.	[16,212,217,219,220,222,223]
**Western Diet (WD)**	Higher risk of preschool wheezing and asthma.	[158,224,231,238]	Deteriorating effect on pulmonary function.	[231,236,237]
**Food Supplements**				
**Polyunsaturated Fatty Acids**	Inconclusive evidence on the protective role on preschool wheezing asthma prevention.	[250,251,252]	No effective role in treating asthma.	[239]
**Antioxidants**	Is shown to protect against preschool wheezing and asthma.	[224,239,275]	No effective role in treating asthma.	[277]
**Vitamin D**	Inconclusive evidence on the protective role on preschool wheezing asthma prevention.	[157,286,287,288,290]	Possible protective role on asthma exacerbations.	[298]
**Probiotics, Prebiotics and Synbiotics**				
	No effective role in preventing asthma.	[308,310]	No effective role in preventing asthma.	[305,316]

## Data Availability

Not applicable.
